# Methionine Sources Differently Affect Production of Reactive Oxygen Species, Mitochondrial Bioenergetics, and Growth of Murine and Quail Myoblasts In Vitro

**DOI:** 10.3390/cimb45040174

**Published:** 2023-03-23

**Authors:** Katja Stange, Toni Schumacher, Claudia Miersch, Rose Whelan, Martina Klünemann, Monika Röntgen

**Affiliations:** 1 Institute of Muscle Biology and Growth, Research Institute for Farm Animal Biology (FBN), Wilhelm-Stahl-Allee 2, 18196 Dummerstorf, Germany; 2 Nutritional Physiology and Dietetics, International University of Applied Sciences (IU), Juri-Gagarin-Ring 152, 99084 Erfurt, Germany; 3 Evonik Operations GmbH, Rodenbacher Chaussee 4, 63457 Hanau, Germany

**Keywords:** methionine, HMTBA, muscle, growth, satellite cell, metabolic rate, viability, ROS

## Abstract

An optimal supply of L-methionine (L-Met) improves muscle growth, whereas over-supplementation exerts adverse effects. To understand the underlying mechanisms, this study aims at exploring effects on the growth, viability, ROS production, and mitochondrial bioenergetics of C2C12 (mouse) and QM7 (quail) myoblasts additionally supplemented (100 or 1000 µM) with L-Met, DL-methionine (DL-Met), or DL-2-hydroxy-4-(methylthio)butanoic acid (DL-HMTBA). In both cell lines, all the supplements stimulated cell growth. However, in contrast to DL-Met, 1000 µM of L-Met (C2C12 cells only) or DL-HMTBA started to retard growth. This negative effect was stronger with DL-HMTBA and was accompanied by significantly elevated levels of extracellular H_2_O_2_, an indicator for OS, in both cell types. In addition, oversupplementation with DL-HMTBA (1000 µM) induced adaptive responses in mitochondrial bioenergetics, including reductions in basal (C2C12 and QM7) and ATP-synthase-linked (C2C12) oxygen consumption, maximal respiration rate, and reserve capacity (QM7). Only QM7 cells switched to nonmitochondrial aerobic glycolysis to reduce ROS production. In conclusion, we found a general negative effect of methionine oversupplementation on cell proliferation. However, only DL-HMTBA-induced growth retardation was associated with OS and adaptive, species–specific alterations in mitochondrial functionality. OS could be better compensated by quail cells, highlighting the role of species differences in the ability to cope with methionine oversupplementation.

## 1. Introduction

In postnatal muscle, so-called satellite cells (SCs) and their progeny are the primary mediators of hypertrophic growth, muscle maintenance, and regeneration, and therefore, their number and molecular and functional properties are crucial in muscle development and plasticity [1,2,3]. SC activation, proliferation, and differentiation have a high requirement for ATP and are, thus, essentially dependent on mitochondrial energy production through aerobic metabolism (respiratory chain and citric acid cycle). Basically, SC functional processes and lineage determination are specifically regulated by myogenic genes, e.g., *Pax7*, *MyoD*, *Myf5*, and *MyoG* [4]. However, metabolic signaling by mitochondria, e.g., via the regulation of Ca^2+^ levels, the release of metabolic intermediates, and the production of reactive oxygen species (ROS) [5,6,7], is also known to play a crucial role. Mitochondria-derived signals affect SC gene expression and functionality, especially with regard to SC differentiation and fate [8,9]. ROS, in particular, function under physiological conditions to adjust cellular activity to available bioenergetic resources, e.g., nutrient availability [10]. Interestingly, the functional activity of both mitochondria and SCs is influenced by Ser/Thr kinase mTORC1, which regulates growth and protein synthesis in response to amino acid availability [11,12,13].

Amino acids play an essential role in muscle growth regulation. They provide the substrate needed as a building block for polypeptide synthesis and modulate signaling pathways involved in protein synthesis and breakdown regulation [14,15,16]. Thus, as with other amino acids, the essential sulfur-containing amino acid methionine is a component of muscle tissue proteins, and its insufficient uptake from the diet leads to reduced protein synthesis by inhibiting mRNA translation [17]. Methionine is often limiting in commercial livestock diets and is, therefore, one of the most added amino acids in the feed industry. In addition, methionine plays an important role in mitochondrial function, the synthesis of polyamines, DNA and protein methylation, insulin sensitivity, cellular redox-buffering systems, and creatine synthesis, making it essential for normal energy and fuel metabolism [15,18,19]. Thus, changes in L-methionine (L-Met) availability might exert a remarkable impact on cellular bioenergetics, ROS status, and activities such as growth. For the balancing of nutritional methionine supply, several sources of the amino acid are used as dietary supplements to improve performance and health in humans and animals [20,21,22,23].

An optimal provision of methionine has been shown to increase organisms’ detoxifying and antioxidant abilities [24,25], which has been linked to positive effects on immune function [26,27]. In humans, methionine supplementation is only recommended under conditions of skeletal muscle wasting and for weightlifters to promote protein synthesis [28,29]. However, synthetic methionine sources, mainly DL-methionine (DL-Met), L-Met, and DL-2-hydroxy-4-(methylthio)butanoic acid (DL-HMTBA, a methionine precursor), are most heavily supplemented in the diets of farm animals, such as high-yielding dairy cows, pigs, and poultry, as well as in fish and shrimp [23]. In these animals, supplementation with methionine has been shown to improve growth performance, carcass quality (reduced fat content), and protein synthesis efficiency [30,31,32,33].

However, uptake of excessive methionine for extended periods is known to inhibit muscle protein synthesis [34] and cell growth [35] and to have adverse effects that may result from increased oxidative stress (OS), followed by, for example, damage to genomic and mitochondrial DNA and increased lipid and protein oxidation [36,37]. As predominant generators of ROS in skeletal muscle cells, mitochondria are particularly vulnerable to injury by high and persistently elevated ROS levels [38]. Defective mitochondria exhibit disturbed energy production and increased OS, both negatively affecting cellular vitality and functions [5]. Progressive mitochondrial dysfunction has also been shown to be involved in the process of aging and in various pathologies, such as diabetic myopathy and degenerative diseases [19,39]. Accordingly, in rodents, methionine restriction has been demonstrated to decrease mitochondrial ROS production and oxidative damage to mitochondrial DNA [40,41] and to increase longevity [42]. The detailed mechanisms for these observed methionine effects, however, have not yet been fully identified.

**Figure 1:**
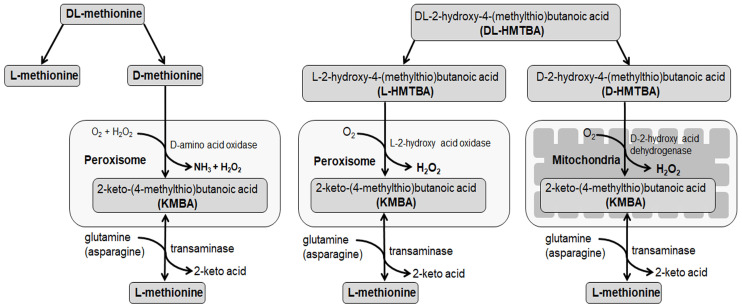
Schematic overview on methionine isomers and precursor transformation pathways.

Of the synthetic methionine sources, DL-HMTBA’s transformation to L-Met (in contrast to that of DL-Met) leads to an additional increase in hydrogen peroxide (H_2_O_2_) production in mitochondria, as well as in peroxisomes (Figure 1). DL-HMTBA is a precursor of methionine by replacing the amino group with a hydroxyl group on the alpha carbon. Consequently, until its conversion to L-Met, DL-HMTBA resembles an organic acid. DL-HMTBA needs to be converted to L-Met for biological utilization, which consists of two enzymatic steps. DL-HMTBA is stereospecifically oxidated to DL-2-keto-4-(methylthio)butanoic acid (KMBA) by peroxisomal L-2-hydroxy acid oxidase localized in peroxisomes or by D-2-hydroxy acid dehydrogenase localized in mitochondria [43]. Subsequently, the common intermediate KMBA is converted to L-Met by transamination, which is a ubiquitous step [44]. D-Met (from DL-Met) is oxidatively deaminated by D-amino acid oxidase in peroxisomes, and the resulting KMBA is transaminated to L-Met [45]. We can, thus, hypothesize that DL-HMTBA, if applied at the same concentration as L-Met or DL-Met, induces higher ROS production and OS, particularly in mitochondria.

OS, defined as the “disruption of redox signaling and control”, is known to influence the replicative capacity of SCs negatively, thereby resulting in premature senescence and apoptosis [46]. In addition, the oxidative damage of proteins enhances their susceptibility to proteolysis [47]. The complex antioxidant system of skeletal muscle cells is able to scavenge and neutralize ROS [5]. Nevertheless, reduced antioxidant capacity or increased OS can overwhelm their enzymatic and nonenzymatic defense mechanisms. Oversupplementation with L-Met, DL-Met, and, in particular, DL-HMTBA via the increased production of ROS might, thus, have negative consequences on mitochondrial bioenergetics and on the functionality of cells with high energy demand, such as SCs and their progeny. Additionally, species-dependent effects cannot be excluded, as most studies are conducted in mammalian species, ignoring adaptive effects of other phylogenetic lines.

In order to achieve positive effects of L-Met on muscle growth and health, the selection of the optimal level, as well as of the best methionine source, is crucial. Specifically, oversupplementation has been shown to exert adverse effects, but the underlying mechanisms are not well understood. For DL-HMTBA, which is often used as a methionine supplement for farm animals, we hypothesize that its transformation to L-Met results in higher ROS production. Therefore, the aim of the present study is to explore the effects of optimal (100 µM) or high (1.000 µM) L-Met, DL-Met, and DL-HMTBA supplementation on C2C12 murine and QM7 quail myoblasts. C2C12 and QM7 are often-used mammalian and avian skeletal muscle model cell lines whose functional behavior corresponds to that of cycling SCs [48]. Specifically, we investigate growth parameters, viability, H_2_O_2_ production, and mitochondrial bioenergetics responses.

## 2. Materials and Methods

### 2.1. Cell Cultures

The mouse and quail skeletal muscle cell lines C2C12 (CRL-1772) and QM7 (CRL-1962) were obtained from the American Type Culture Collection (ATCC, Manassas, VA, USA), and recommended basal media was used. Cells were cultured according to the supplier’s recommendations. C2C12 cells were cultured in the following growth medium (GM): Dulbecco’s modified Eagle’s medium (DMEM), 10% fetal bovine serum (FBS), and 10 U/mL penicillin/streptomycin. QM7 cells were cultured in the following GM: Medium 199 (EBSS) with 10% tryptose phosphate broth, 10% FBS, and 10 U/mL penicillin/streptomycin. Both cell lines were passaged using HyQTAse three times a week before they reached about 70% confluency. Cell number was determined using a Countess Automated Cell Counter (Thermo Fisher, Darmstadt, Germany). All cultures were incubated at 37 °C under a humidified atmosphere of 95% air and 5% CO_2_. After culture establishment, the cells were split up into groups, fed with regular GM or regular GM, and supplemented with 100 or 1000 µM of L-Met, DL-Met, or DL-HMTBA (Evonik Nutrition and Care GmbH, Hanau, Germany). Media (DMEM and Medium 199) and antibiotics (penicillin/streptomycin) were purchased from PAN Biotech (Aidenbach, Germany), tryptose phosphate broth and FBS from Gibco life technologies (Darmstadt, Germany), and HyQTAse from Thermo Fisher Scientific (Bremen, Germany).

### 2.2. Impedance-Based Recording and Analysis of Real-Time Kinetic Growth Curves

**Figure 2:**
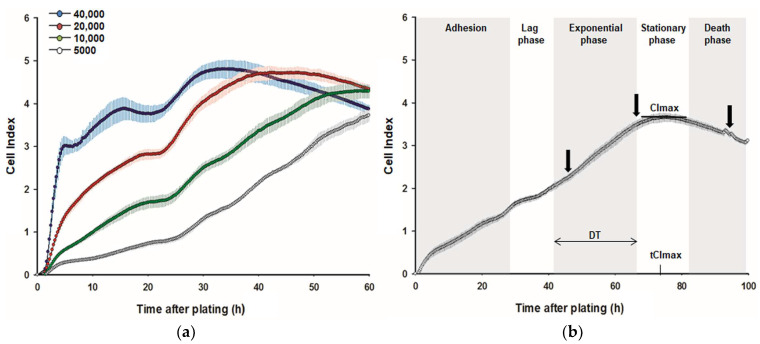
xCELLigence-system-based real-time monitoring of C2C12 cell growth kinetics. (**a**) C2C12 cells were seeded at densities of 5000, 10,000, 20,000, or 40,000 cells per well to select an appropriate cell number for proliferation assays. The results indicated that a cell density of 5000 per well could be chosen as a standard. Representative growth curves are shown. (**b**) Representative original kinetic growth curve and characteristic growth phases for C2C12 cells seeded at a density of 5000 per well. During the exponential phase, the doubling time (DT) was calculated. At the end of the exponential phase (transition to stationary phase), the maximum Cell Index (CImax) and the time to achieve the CImax (tCImax) were determined. The black arrows symbolize the time points for the determination of DNA and protein amount, as well as viability and metabolic activity (WST-1).

Real-time kinetic growth curves for C2C12 and QM7 cells were recorded over a period of at least 96 h by means of an xCELLigence system (RTCA-SP, Omni Life Sciences, Bremen, Germany). An RTCA Analyzer measured electrical impedance changes that occurred across interdigitated microelectrodes integrated on the bottom of a specialized 96-well plate (E-Plate 96) resulting from alterations in the number or dimension of the attached cells. Data were sent to an RTCA control subunit that used RTCA Software (version 2.0) for the real-time display of results as a dimensionless Cell Index (CI) and for further growth parameter calculations [49]. To select the seeding density most suitable to investigate growth properties before and after supplementation with L-Met, DL-Met, or DL-HMTBA, cell numbers of 5000, 10,000, 20,000, or 40,000 cells per well were tested, as shown for C2C12 cells in Figure 2a. Based on these trials, cell densities of 5000 or 10,000 per well were selected for further experiments with C2C12 or QM7 cells, respectively. Before seeding cells, the background impedance of an E-Plate was determined with 100 µL GM or 100 µL GM containing L-Met, DL-Met, or DL-HMTBA per well. Subsequently, cells suspended in 100 µL GM were added to give a final volume of 200 µL per well. Then, the RTCA Analyzer unit was placed into a CO_2_ incubator, and the CI was monitored every 15 min until the end of the experiments. Figure 2b shows characteristic growth phases with C2C12 cells as an example. During the first 25 h, the growth curves mainly reflected the processes of cell attachment and cell spreading (adhesion). Thereafter, cells underwent an obvious lag phase before entering the exponential (logarithmic) growth phase, followed by transition into the stationary growth phase in which the maximum CI (CImax) was achieved. Because of waste toxicity and nutrient starvation, the CI started to decrease during the subsequent death phase. To describe and compare the growth behavior of C2C12 and QM7 cells, in addition to the CImax, the time needed to attain the CImax (tCImax) and the doubling time (DT) were determined from the established growth curves. All samples were plated in quadruplicate, and 3 independent experiments were performed.

### 2.3. Assessment of Cell Viability and Metabolic Activity

Cell viability was determined with a WST-1 (water-soluble tetrazolium) assay (Roche Diagnostics GmbH, Mannheim, Germany). The assay was based on the ability to reduce the light-red-colored tetrazolium salt WST-1 (4-[3-(4-iodophenyl)-2-(4-nitrophenyl)-2H-5-tetrazolio]-1,3-benzol-disulfonate) into a purple formazan reaction product. C2C12 or QM7 cells suspended in GM were seeded at densities of 5000 or 10,000 cells/well, respectively, into 96-well flat-bottomed plates and cultured for 48, 72, or 96 h with or without supplementation. All samples were plated in quadruplicate per treatment, and 5 independent experiments were performed. At the respective time points, 20 µL of WST-1 reagent was added directly into the wells containing cells in 200 µL of cell suspension. The 96-well plate was then incubated for 1 h at 37 °C. After incubation, 100 µL of reaction mix was removed and pipetted into a fresh standard plate. The absorbance was determined at a wavelength of 437 nm and reference wavelength of 690 nm using a microplate reader. The measured absorbance was corrected for background (blanks containing the respective media only) and was considered to reflect the metabolic activity of the muscle cells.

### 2.4. Determination of DNA and Protein Concentration

DNA was measured fluorometrically with a Fluorescence Reader FLx800 (Bio-Tek Instruments GmbH, Bad Friedrichshall, Germany) against a standard of calf thymus DNA (Sigma-Aldrich, St. Louis, MO, USA) after use of Hoechst 33258 (Sigma-Aldrich, St. Louis, MO, USA) according to Rago [50]. Protein concentration was determined according to Peterson [51]. C2C12 or QM7 cells were cultured in a 96-well flat-bottomed microtiter plate (5000 or 10,000 cells per 200 µL GM/well, respectively) with or without supplementation, and the amounts of DNA and protein were determined after 48, 72, and 96 h. All samples were plated in quadruplicate per treatment, and 5 independent experiments were performed.

### 2.5. Hydrogen Peroxide Assay

Myoblasts were seeded at a density of 25,000 cells/well in a 24-well plate containing GM and the respective supplements (L-Met, DL-Met, or DL-HMTBA). Then, by use of an OxiSelect Hydrogen Peroxide/Peroxidase Assay Kit (Hoelzel Diagnostika Handels GmbH, Cologne, Germany, [52]), the H_2_O_2_ levels in the supernatants were measured after 48, 72, and 96 h. All samples were plated in quadruplicate per treatment, and 5 independent experiments were performed. The assay was based on the H_2_O_2_- and horseradish peroxidase (HRP)-mediated oxidation of nonfluorescent 10-acetyl-3, 7-dihydroxyphenoxazine (ADHP) for the production of highly fluorescent resorufin and was performed according to the manufacturer’s instructions.

### 2.6. Determination of Oxygen Consumption Rate (OCR) and Mitochondrial Bioenergetics

First, cell numbers between 5000 and 60,000 cells/well were tested to identify optimal assay conditions. For our investigations, 25,000 cells/well were seeded in quadruplicate per treatment; 5 independent experiments were performed. Cells were incubated in GM supplemented with L-Met, DL-Met, or DL-HMTBA for 48 h; detached with HyQTAse; and washed twice in Dulbecco‘s phosphate-buffered saline (DPBS, PAN Biotech). The oxygen consumption rate (OCR) of C2C12 and QM7 cells was measured as described previously using a multimode reader (VICTOR3, PerkinElmer, Waltham, MA, USA) [53]. In brief, cells were seeded on fluorophore-coated 96-well OxoPlates (Precision Sensing GmbH, Regensburg, Germany) in measuring medium (MM, DMEM without NaHCO_3_ and phenol red, 5.5 mM glucose, 1 mM Na-pyruvate, 4 mM stable glutamine, and 5 mM HEPES, pH 7.3, osmolarity 290 mosmol/L, PAN Biotech). Fluorescence intensity increased when cells survived or grew [54]. The basal OCR (OCRbas) was measured for 15 min. Subsequently, every 15 min, the inhibitors oligomycin, FCCP (carbonyl cyanide p trifluoromethoxyphenyl-hydrazone), and antimycin A1 (all purchased from Sigma Aldrich, final concentration 10 µM) were added. Fluorescence values were converted to OCR/well according to the manufacturer’s protocol.

### 2.7. Statistical Analysis

Statistical analyses were performed using SigmaPlot for Windows, version 11.0 (Systat Software Inc.). For all parameters, descriptive statistics was performed, and data are presented as means ± standard error (SE). Samples were tested for normality (Shapiro-Wilk) and equal variance (Brown-Forsythe). The data were considered as approximately normal and were analyzed with ANOVA (analysis of variance). The ANOVA models for the parameters of DNA, protein, viability and metabolic activity, and hydrogen peroxide production contained the fixed factors group (levels: L-Met, DL-Met, and HMTBA), supplementation (levels: 0 µM, 100 µM, and 1000 µM; hydrogen peroxide production data without 100 µM), and time (levels: 48 h, 72 h, and 96 h), as well as all interactions between the fixed factors.

The ANOVA models for the CImax, DT, tCImax, and OCR data contained the fixed factors group (levels: L-Met, DL-Met, and DL-HMTBA), supplementation (levels: 0 µM, 100 µM, and 1000 µM; OCR data only 1000 µM), and the interactions of groups with supplementation. Least-squares means (LSMs) and their standard errors (SE) were computed for each fixed effect in the models, and all pairwise differences of these LSMs were tested using the Holm–Sidak method, a procedure for pairwise multiple comparisons. If the normality test or the equal variance test failed, Kruskal–Wallis ANOVA on Ranks was performed with a Tukey test (equal sampling) or Dunn’s method (unequal sampling) as a pairwise comparison procedure. In addition, the partitioned analyses of the LSMs for the two-way interactions of group with supplementation (i.e., test of a group within the levels of supplementation and test of supplementation within the levels of group) were performed. For the comparison of two groups, the statistical significance was assessed using Student’s *t*-test. Effects and differences were considered significant if *p* ≤ 0.05.

If no significant differences were detected between groups, values were combined if appropriate (as for DNA and protein (methionine supplements combined), WST-1 (concentrations of each supplement combined), and hydrogen peroxide assay (time points combined)).

## 3. Results

### 3.1. Effect of L-Met, DL-Met, and DL-HMTBA on Cellular Growth Kinetics and Proliferation

**Figure 3:**
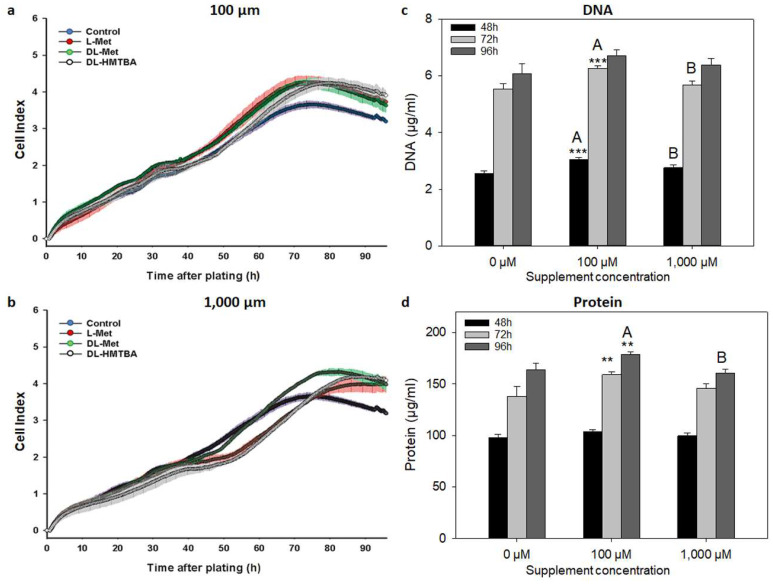
Effects of L-Met, DL-Met, and DL-HMTBA on growth kinetics (**a**,**b**) and DNA (**c**) and protein concentrations (**d**) of C2C12 cells. Representative kinetic growth curves for C2C12 cells (5000 cells/well) supplemented with 100 µM (**a**) or 1000 µM (**b**) of L Met, DL-Met, or DL-HMTBA are shown in comparison with controls without supplement. For the evaluation of DNA (**c**) and protein (**d**) values, data for all methionine supplements were pooled since there were no statistical differences between them. Supplementation with methionine sources stimulated cell growth and increased DNA and protein concentrations at a concentration level of 100 µM. ** *p* ≤ 0.01 and *** *p* ≤ 0.001 vs. control; A and B uppercase letters show significant differences between supplements within a concentration value (*p* ≤ 0.05); N = 3 (for growth curves) and N = 15 (for DNA and protein).

**Figure 4:**
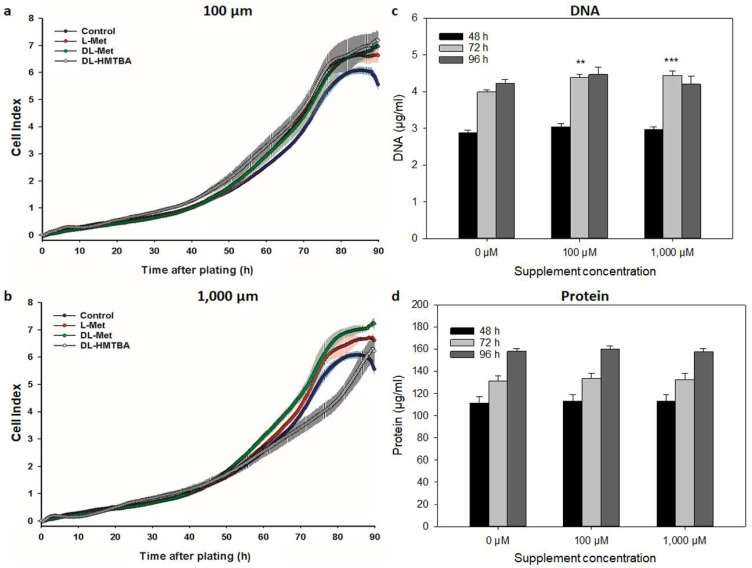
Effects of L-Met, DL-Met, and DL-HMTBA on growth kinetics (**a**,**b**) and DNA (**c**) and protein concentrations (**d**) of QM7 cells. Representative kinetic growth curves for QM7 cells (10,000 cells/well) supplemented with 100 µM (**a**) or 1000 µM (**b**) of L Met, DL-Met, or DL-HMTBA are shown in comparison with controls without supplement. For the evaluation of DNA (**c**) and protein (**d**) values, data for all methionine supplements were pooled since there were no statistical differences between them. Supplementation with methionine sources stimulated cell growth at both tested concentrations. DNA concentration in QM7 cells was significantly increased after methionine supplementation for 72 h. ** *p* ≤ 0.01 and *** *p* ≤ 0.001 vs. control; N = 3 (for growth curves) and N = 15 (for DNA and protein).

Real-time impedance measurements based on xCELLigence technology that displayed results as a dimensionless CI for further growth parameter calculations allowed us to determine the effects of 100 or 1000 µM of respective methionine sources on C2C12 and QM7 cell growth kinetics. The kinetic growth parameters of CImax, tCImax, and DT are summarized in Table 1 for each cell line and the respective treatments. Representative growth curves of C2C12 and QM7 cells supplemented with 100 µM or 1000 µM of L-Met, DL-Met, or DL-HMTBA and their respective controls are depicted in Figure 3a,b (C2C12) and Figure 4a,b (QM7).

**Table 1:** Kinetic growth parameters of murine C2C12 and avian QM7 cells after supplementation with L-Met, DL-Met, and DL-HMTBA.

			Growth Parameters							
Cell Line	Supplement	Concentration	CImax		tCImax			DT		
		[µM]	(AU)		(h)			(h)		
C2C12	Control	0	3.69 ± 0.11		74.3 ± 1.3			30.9 ± 0.4		
	L-Met	100.0	4.11 ± 0.15 *		75.2 ± 0.9		a	26.8 ± 0.9		
	DL-Met	100.0	4.10 ± 0.11		73.0 ± 0.5			28.2 ± 1.0		
	DL-HMTBA	100.0	4.22 ± 0.13 **		75.2 ± 1.4		a	27.2 ± 1.3		
	L-Met	1000.0	4.00 ± 0.12		88.0 ± 1.2 ***	A	b	28.6 ± 1.4		
	DL-Met	1000.0	4.15 ± 0.06 *		73.8 ± 0.7	B		27.3 ± 0.6		
	DL-HMTBA	1000.0	4.09 ± 0.08		93.3 ± 2.7 ***	C	b	27.1 ± 0.6		
QM7	Control	0	6.04 ± 0.12		84.5 ± 0.8			15.7 ± 0.6		
	L-Met	100.0	6.64 ± 0.16 **		88.7 ± 0.6 *			14.0 ± 0.2 *		
	DL-Met	100.0	6.96 ± 0.08 **		89.0 ± 0.6 *			14.1 ± 0.1 *		
	DL-HMTBA	100.0	7.21 ± 0.19 ***		88.8 ± 0.7 *		a	15.8 ± 0.1		a
	L-Met	1000.0	6.70 ± 0.08		87.3 ± 1.2	A		14.3 ± 0.2	A	
	DL-Met	1000.0	7.23 ± 0.12 ***		87.8 ± 1.4	A		13.9 ± 0.1 *	A	
	DL-HMTBA	1000.0	6.91 ± 0.26		94.5 ± 0.9 **	B	b	19.4 ± 0.3 ***	B	b

Kinetic growth curves were recorded by use of an xCELLigence system, and the following parameters were calculated: (1) the maximum Cell Index (CImax) as a measure of maximum growth capacity, (2) the time to achieve the CImax (tCImax), and (3) the doubling time (DT), which is the period of time required for the CI to double during exponential growth. CImax, tCImax, and DT values are given as LSM ± SE, (N = 3). Significantly increased or decreased parameters (compared to control) are marked with green or red, respectively, for a better overview. * *p* ≤ 0.05, ** *p* ≤ 0.01, and *** *p* ≤ 0.001 vs. control; uppercase letters (A, B, and C) show significant differences between supplements within a concentration value; lowercase letters (a and b) show significant concentration effects for a given supplement (*p* ≤ 0.05).

Contributing to their different origins, the control groups of C2C12 and QM7 reached the CImax at differential levels of cell index of 3.7 and 6.0, respectively. These values were reached after 74 h (C2C12) and 85 h (QM7), representing the tCImax. As seen from Table 1 and Figure 3a,b and Figure 4a,b, all methionine supplements significantly stimulated cell growth compared with control cells. While numerically similar to L-Met with 100 µM of supplementation, DL-Met only reached a significant stimulating effect at 1000 µM in C2C12 cells according to the CImax (Table 1). QM7 cells seemed to be more susceptible to methionine supplementation since either of the supplements significantly increased the CImax above control levels at a concentration of 100 µM. DL-Met further enhanced the CImax at the highest concentration of 1000 µM in QM7. The time to reach the CImax was increased due to a prolongation of the lag phase (Figure 3 and Figure 4a,b) in C2C12 cells after treatment with either L-Met or DL-HMTBA at 1000 µM, as well as in QM7 after supplementation with 100 µM for any methionine source. At the highest concentration, only DL-HMTBA significantly increased the tCImax in QM7 cells.

The DT of C2C12 cells (31 h) was not affected by the individual supplements. However, the DT was generally reduced to 27.5 ± 0.5 h (*p* < 0.001 vs. control) in supplemented C2C12 cells. In QM7 cells, the DT was shortened by treatment with L-Met (100 µM) and DL-Met (100 and 1000 µM) and increased by 1000 µM of DL-HMTBA. Thus, as revealed by prolongation of the lag phase (C2C12 cells), the DT (QM7 cells only), and the tCImax, L-Met (C2C12 cells only) and DL-HMTBA started to retard cell growth at a concentration of 1000 µM. In C2C12 cells, the negative effect of DL-HMTBA was significantly stronger than that of L-Met.

To characterize the cellular growth processes further, we determined the DNA and protein contents of control and supplemented C2C12 and QM7 cells at 48, 72, and 96 h of treatment. In C2C12 cells (Figure 3c,d), 100 µM of supplement increased the cellular DNA (48 and 72 h) and protein content (72 and 96 h) significantly above control values, independent of supplemented methionine source. Additionally, 1000 µM of supplement decreased DNA and protein concentrations compared with 100 µM. In QM7 cells (Figure 4c,d), 100 and 1000 µM of supplement significantly increased cellular DNA (72 h) concentration.

### 3.2. Effect of L-Met, DL-Met, and DL-HMTBA on Viability and Metabolic Activity

**Figure 5:**
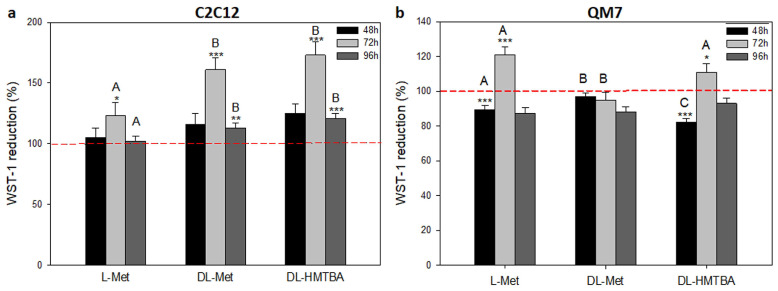
Effects of L-Met, DL-Met, and DL-HMTBA on cell viability and metabolic activity of C2C12 (**a**) and QM7 cells (**b**). C2C12 or QM7 cells were seeded at densities of 5000 or 10,000 cells per well, respectively. Metabolic activity was determined via WST-1 assay after 48, 72, or 96 h after application of 100 or 1000 µM of L-Met, DL-Met, or DL-HMTBA; tested concentrations were pooled since there were no statistical differences between them. In C2C12 cells, L-Met, DL-Met, and DL-HMTBA significantly increased cellular activity compared to unstimulated control cells. Contrastingly, in QM7 cells, L-Met and DL-HMTBA decreased metabolic activity after 48 h but increased it after 72 h. * *p* ≤ 0.05, ** *p* ≤ 0.01, and *** *p* ≤ 0.001 vs. control; A, B, and C uppercase letters show significant differences between supplements within a concentration value (*p* ≤ 0.05); N = 10.

A possible interrelationship between growth effects and the viability and metabolic activity was determined using an WST-1 assay on C2C12 and QM7 cells supplemented with L-Met, DL-Met, or DL-HMTBA. WST-1 reduction was measured at 48, 72, and 96 h after addition of the respective supplements (Figure 5). In C2C12 cells (Figure 5a), supplementation with methionine sources increased metabolic activity above control values at 72 (all methionine sources) and 96 h (DL-Met and DL-HMTBA) after seeding, with stronger effects of DL-Met and DL-HMTBA. In QM7 cells, however, DL-Met supplementation had no effect on WST-1 reduction. In contrast, L-Met and DL-HMTBA led to time-dependent changes in metabolic activity (Figure 5b). At 48 h, a significant reduction was found, whereas an increase was observed 72 h after seeding.

### 3.3. Effect of L-Met, DL-Met, and DL-HMTBA on Extracellular Hydrogen Peroxide Production

We assumed that changes in metabolic activity as found in the WST-1 assay could be associated with alterations in ROS generation. For this reason, we tested the impact of high (1000 µM) concentrations of L-Met, DL-Met, and DL HMTBA on H_2_O_2_ concentration in the culture medium.

**Figure 6:**
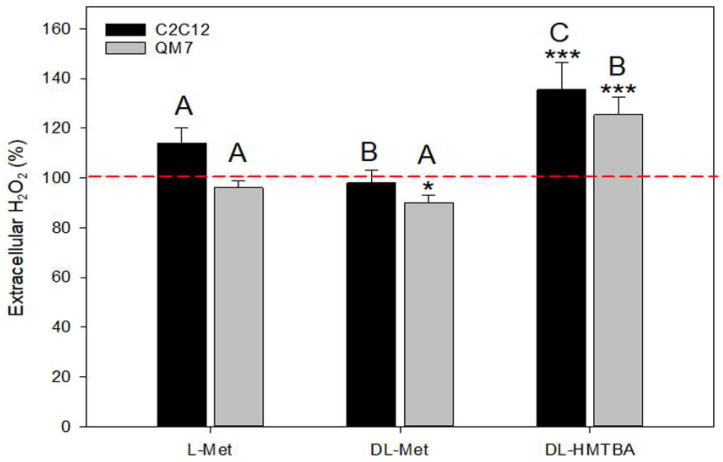
Extracellular H_2_O_2_ concentration in medium for C2C12 and QM7 cells after supplementation with 1000 µM of L-Met, DL-Met, or DL-HMTBA. Cells were seeded at a density of 25,000 cells per well. The concentration of extracellular H_2_O_2_ was determined at 48, 72, and 96 h after supplementation, and data from all time points were pooled, as there was no significant time dependency detected. The H_2_O_2_ concentration in the control medium was set to 100%, and the H_2_O_2_ concentration after supplementation is given as a percentage of the control. DL-Met significantly reduced extracellular H_2_O_2_ levels in QM7 cells. DL-HMTBA enhanced H_2_O_2_ secretion in both tested cell lines. * *p* ≤ 0.05 and *** *p* ≤ 0.001 vs. control; A, B, and C uppercase letters show significant differences between supplements within a concentration value (*p* ≤ 0.05); N = 30.

The data are summarized in Figure 6 and show that the direction and strength of the observed changes in medium H_2_O_2_ concentration depended on the type of supplement. L-Met induced no significant effect on extracellular H_2_O_2_ levels in either of the tested cell types. In QM7 cells, extracellular H_2_O_2_ concentration was significantly decreased after DL-Met supplementation, whereas DL-HMTBA supplementation led to significantly elevated peroxide levels in both cell lines.

### 3.4. Metabolic Rate and Mitochondrial Function after Treatment with L-Met, DL-Met, or DL HMTBA

**Figure 7:**
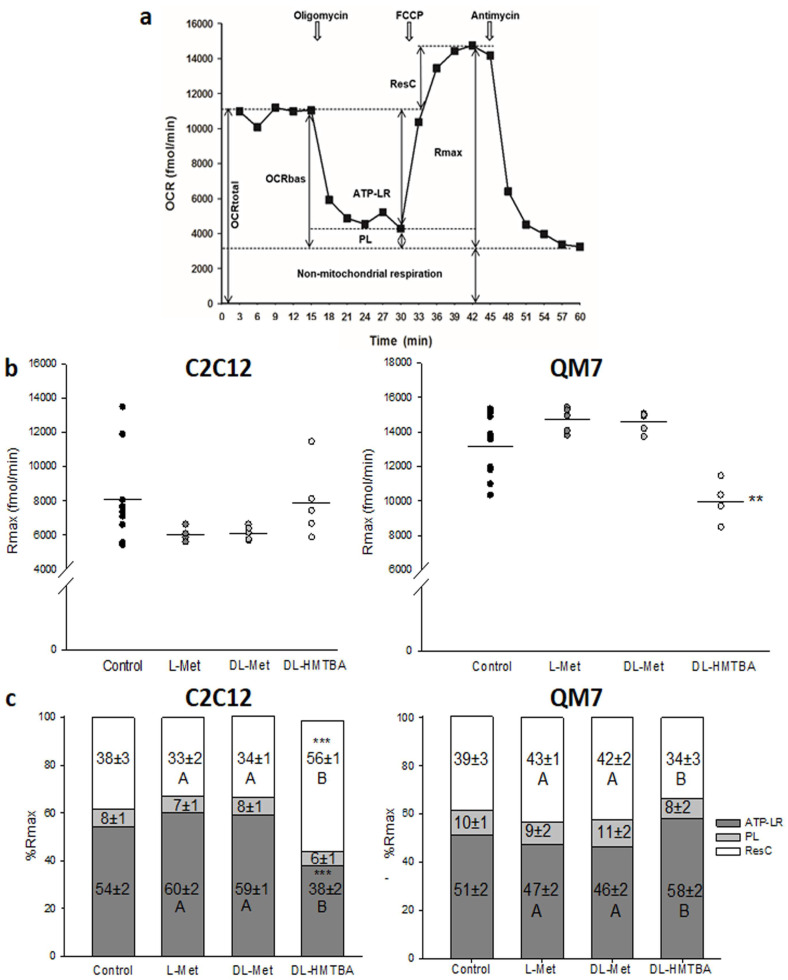
Measurement of bioenergetic parameters in C2C12 and QM7 cells after supplementation with 1000 µM of L-Met, DL-Met, or DL-HMTBA. (**a**) Schematic view of oxygen consumption rates (OCRs) measured under basal conditions (OCRbas) and in response to indicated mitochondrial inhibitors. The arrows show the sequential injection of oligomycin, FCCP, and antimycin A1 (10 µM each). Parameters calculated are also shown. ATP synthase-linked respiration (ATP-LR) and the respiration rate attributable to proton leak (PL) were calculated using OCRbas and oligomycin-insensitive respiration. FCCP uncoupled oxidative phosphorylation and disrupted the electrochemical gradient (proton motif force; Δψm). It was employed to determine the maximal OCR (Rmax) of the cells. The injection of antimycin A1, a complex III inhibitor, allowed for the measurement of nonmitochondrial oxygen consumption by shutting down mitochondrial respiration. The mitochondrial reserve capacity (ResC) was calculated as the difference between the Rmax and OCRbas. The total OCR was the sum of the OCRbas and the nonmitochondrial respiration. (**b**,**c**) Total values of Rmax (**b**) and proportions (**c**) of ATP-linked respiration (ATP-LR), proton leak (PL), and reserve capacity (ResC) on maximum FCCP-induced oxygen consumption rate (Rmax = 100%; **c**) in C2C12 and QM7 cells treated with 1000 µM of L-Met, DL-Met, or DL-HMTBA. Unstimulated cells served as controls. DL-HMBTA significantly reduced the Rmax in QM7 cells. In C2C12 cells, no significant changes in the Rmax were induced by methionine. Nevertheless, the proportional composition of the Rmax was significantly altered by supplementation with 1000 µM of DL-HMTBA in C2C12 cells, as seen by the elevated ResC. ** *p* ≤ 0.01 and *** *p* ≤ 0.001 vs. control; A and B uppercase letters show significant differences between supplements within a concentration value (*p* ≤ 0.05). N = 15 (control); N = 5 (supplements).

To gain information on the possible effects of L-Met, DL-Met, and DL-HMTBA treatment on bioenergetic processes, we analyzed oxygen consumption rate and mitochondrial function in C2C12 and QM7 myoblasts after pretreatment with 1000 µM of these supplements (Figure 7, Table 2). Unsupplemented cells were used as controls, and a typical experimental procedure is presented in Figure 6a. The following parameters could be derived from differences in the OCR between experimental phases: (1) nonmitochondrial respiration, (2) OCRbas (current mitochondrial OCR) with its components of ATP-synthase-linked respiration (ATP-LR) and proton leak (PL), and (3) mitochondrial reserve capacity (ResC). The latter could be calculated after the induction of maximum mitochondrial respiration (Rmax) by use of the uncoupling agent FCCP. Nonmitochondrial respiration could be assessed after completely inhibiting mitochondrial respiration at the end of the experiment with antimycin. As shown in Table 2, the absolute value of nonmitochondrial respiration was not affected by supplementation with methionine sources in C2C12 cells. However, in QM7 cells, we found a marked increase in nonmitochondrial respiration after treatment with DL-HMTBA.

**Table 2:** Effects of supplementation with 1000 µM of L-Met, DL-Met, or DL-HMTBA on bioenergetics and mitochondrial function of C2C12 and QM7 cells.

Cell Line	Group		Nonmitochondrial Respiration	Mitochondrial Respiration			
		Concen-tration		OCRbas		ATP-LR	PL
		[µM]	(fmol/min)	(fmol/min)		(fmol/min)	(fmol/min)
C2C12	Control	0	1682 ± 92	4832 ± 358		4253 ± 355	579 ± 48
	L-Met	1000	1736 ± 39	4039 ± 129		3630 ± 153	409 ± 80
	DL-Met	1000	1693 ± 51	4048 ± 105		3583 ± 32	465 ± 77 #
	DL-HMTBA	1000	1808 ± 255	3482 ± 398 ***		3012 ± 387 ***	448 ± 105
QM7	Control	0	2925 ± 145	7962 ± 218		6647 ± 278	1315 ± 150
	L-Met	1000	2419 ± 98	8325 ± 209	A	6964 ± 426	1360 ± 296
	DL-Met	1000	2394 ± 111	8394 ± 286	A	6744 ± 435	1651 ± 238
	DL-HMTBA	1000	4058 ± 281 **	6544 ± 248 **	B	5773 ± 344	771 ± 142

The following parameters were computed: nonmitochondrial respiration, basal OCR (OCRbas = current mitochondrial OCR) with its components of ATP-synthase-linked respiration (ATP-LR) and proton leak (PL), and mitochondrial reserve capacity (ResC, not shown here). Results are given as LSM ± SE (N = 5). Significantly increased or decreased parameters (compared to control) are marked with green or red, respectively, for a better overview. ** *p* ≤ 0.01 and *** *p* ≤ 0.001 vs. control; uppercase letters (A and B) show significant differences between supplements (*p* ≤ 0.05); # shows a significant difference between control and supplemented cells (all supplements combined together). N = 15 (control); N = 5 (supplements).

In unsupplemented C2C12 and QM7 cells, the proportion of nonmitochondrial respiration (%nmR) accounted for 27 ± 1% of the measured OCR, and it was increased to 36 ± 1% (*p* < 0.001) after supplementation with DL-HMTBA. The %nmR differed between C2C12 and QM7 cells supplemented with L-Met and DL-Met. Treatment with both supplements let to a higher %nmR in C2C12 compared with QM7 cells (30 ± 1% vs. 22 ± 1%; *p* < 0.001).

The Rmax reflected the total oxidative capacity of mitochondria and amounted to 8071 ± 826 fmol/min (C2C12) and 13,151 ± 563 fmol/min (QM7) under control conditions (Table 2 and Figure 7b). There was no significant effect of any methionine source on the total value of the Rmax in C2C12 cells, whereas in QM7 cells, supplementation with DL-HMTBA reduced the Rmax (9933 ± 484 fmol/min) compared with control values (*p* < 0.004) (Table 2 and Figure 7c).

In both cell types, we observed a significant decrease in the OCRbas after supplementation with DL-HMTBA, and this was accompanied by a significant reduction in ATP-LR in C2C12 cells (Table 2). In QM7 cells, instead, the decreasing effect of high-dosage DL-HMTBA supplementation on ATP-LR was not observed. In addition, PL was generally lowered when C2C12 cells were supplemented with a methionine source.

Figure 6c summarizes the effects of all the methionine sources (1000 µM) on mitochondrial bioenergetics by showing the proportions of ATP-LR, PL, and ResC in relation to the Rmax (%ATP-LR, %PL, and %ResC) in nontreated and treated C2C12 and QM7 cells. The %ResC amounted to 38% (3239 ± 507 fmol/min) and 39% (5189 ± 466 fmol/min) in nontreated C2C12 and QM7 cells, respectively. The control values for %ATP-LR and %PL were 54%/51% and 8%/10%, respectively. In both cell types, no change in the %PL was induced by supplementation with methionine sources. However, compared with control, L-Met-, and DL-Met-treated C2C12 cells, DL-HMTBA reduced the %ATP-LR and thus, increased the %ResC in C2C12 cells. In contrast, treatment of QM7 cells with DL-HMTBA led to higher %ATP-LR and lower %ResC values compared with L-Met- and DL-Met-supplemented cells.

## 4. Discussion

Nutrition has been shown to impact SC activity, proliferation, and differentiation and, subsequently, muscle growth, maintenance, and ability for regeneration [16,55]. However, little is known about the role of amino acid metabolism in SCs and stem cells in general. Interestingly, in human embryonic and induced pluripotent stem cells, methionine metabolism was shown to regulate maintenance and differentiation [56].

Our analyses were conducted to gain more insights into the impact of different supplemented methionine sources on cellular growth and metabolism, especially regarding oxidative respiration and oxidative stress response. To this aim, we used an xCELLigence system to record real-time kinetic growth curves from C2C12 and QM7 muscle cells cultivated either in medium with recommended methionine levels (control) or in media additionally supplemented with 100 µM or 1000 µM of L-Met, DL-Met, or DL-HMTBA. The calculated CI mainly reflected changes in cell number, as well as increases when more cells attached or spread and decreases when cells detached or died. Thus, CI changes could be used to quantify positive or negative growth effects induced by various methionine sources and concentrations. Moreover, the DT and, thus, the growth rate could be calculated from CI changes during the logarithmic growth phase. As the resolution of the CI was at the overall cellular level, cellular DNA and protein contents, as well as viability and metabolic activity, were determined in parallel experiments.

Generally, growth kinetics differed markedly between rodent C2C12 and quail QM7 cell lines, with a faster proliferation rate (shorter DT) in the latter. Moreover, the CImax was approximately 64% higher in QM7 compared to C2C12 cells. Our findings are in accord with data from [57] showing that avian fibroblasts generally proliferated faster than those from rodents. In addition, compared with similar-sized mammals, birds have a higher metabolic rate [58]. Indeed, under control conditions, the OCRbas of quail QM7 cells was 65% higher than that of mouse C2C12 cells in the present study. This agrees well with the 1.5-fold difference in metabolic rate between mice and quail [58]. It is imperative to consider these differences when interpreting results from experiments with methionine supplementation.

We found cell growth to be stimulated by all the methionine sources in both cell types, which was reflected by higher CImax values and increased cellular DNA amounts compared with controls. Protein synthesis was stimulated in C2C12 cells by methionine supplementation. The utilization of DL-Met and DL-HMTBA mainly depends on their conversion to L-Met, which is then used in various metabolic processes, including the synthesis of DNA and protein and the formation of the methyl donor S-adenosylmethionine and of L-cysteine, creatine, glutathione, and taurine [59,60,61]. The positive effect of L-Met on protein synthesis [62] is known to result not only from providing a substrate for polypeptide synthesis, but also from the activation of translation via p70S6K1 and RPS [63], an effect that has not yet been observed with D-Met or DL-HMTBA [17]. This agrees with the role of specific structural properties needed to induce a full stimulating effect and with investigations showing that the extracellular concentration of essential amino acids, rather than their intracellular availability, acutely regulates skeletal muscle synthesis of protein [64,65].

In addition to protein accretion, cell proliferation is a fundamental process in muscle growth and maintenance [16,55]. In our model, the increased CI in methionine-supplemented cells revealed a pro-proliferative activity for all the methionine sources. In accord, supplementation with 100 µM of any methionine source increased the DNA amount, which is a robust indicator of cell proliferation [66]. In addition, the positive effect of the methionine sources on growth was mainly associated with a further reduction in DT (DL-Met and L-Met) and, thus, an increase in proliferation rate in QM7 cells. We hypothesized that increased growth rate through additional methionine supplementation might result in higher numbers of myogenic cells in muscle, thereby increasing the cell pool available for differentiation into multinucleated myotubes and for hypertrophic myofiber growth.

In parallel to the growth curves, we measured the overall cellular metabolic activity of the cells using an WST-1 reduction assay that reflected, to a great extent, the activity of the mitochondrial enzyme succinate dehydrogenase-complex II of the respiratory chain [67]. Our data showed that, with the exception of DL-Met in QM7 cells, WST-1 reduction and, thus, mitochondrial activity were increased in actively proliferating cells supplemented with methionine sources. As expected, the effect was most prominent during the exponential growth phase of both cell types (after 72 h of cultivation with the methionine sources) but stronger and prolonged in C2C12 compared with QM7 cells. These results point to the different regulation of mitochondrial processes in methionine-supplemented mice and quail cells.

At a concentration of 1000 µM, the growth behavior of C2C12 cells was negatively affected by exposure to L-Met and DL-HMTBA, whereas in QM7 cells, this effect was observed with DL-HMTBA only. Growth curves of 1000 µM of L-Met- or DL-HMTBA-supplemented C2C12 cells showed no increase in the CImax compared to control cells and exhibit an extended lag phase, needing longer than DL-Met-treated cells to reach the CImax. In QM7 cells, only treatment with 1000 µM of DL-HMTBA slowed down growth due to a strongly increased DT. Thus, lowering of the proliferation rate was an important component of the growth-retarding effect of 1000 µM of DL-HMTBA in QM7 cells. We supposed that the growth-decelerating effects of high L-Met (in C2C12 cells only) or high HMTBA levels were related to cellular ROS production.

According to our hypothesis, supplementation with methionine sources, particularly of the synthetic methionine source DL-HMTBA, would increase the intracellular production of ROS, mainly of H_2_O_2_. Under such conditions, cells aim to stabilize normal ROS levels by reducing the production of oxidants or enhancing clearance via the activation of antioxidant defense systems [68]. Nevertheless, the clearance of H_2_O_2_ hinges on the redox-buffering capacity of a cell, which can be overwhelmed by high H_2_O_2_ levels [69,70]. H_2_O_2_ is the main mitochondria-derived ROS, as it is the product of superoxide detoxification by the mitochondrial enzyme manganese superoxide dismutase (MnSOD) [71,72]. According to the results of our WST-1 assays, all the supplements (DL-Met only in C2C12 cells) increased mitochondrial activity, but neither L-Met nor DL-Met increased the H_2_O_2_ secretion of both tested cell types. In contrast, we found a significant elevation in the extracellular H_2_O_2_ concentration after DL-HMTBA treatment. These data are in line with additional H_2_O_2_ production relating to DL-HMTBA’s transformation into L-Met, resulting in overloading of the cellular antioxidant system and the subsequent externalization of H_2_O_2_. Most likely, the mitochondrial glutathione peroxidase/glutathione system became overwhelmed if supplement concentrations became too high [73]. Oversupplementation of L-Met (in C2C12 cells only) and of DL-HMTBA (C2C12 and QM7 cells) also led to a higher and competing demand for methionine/cysteine to form glutathione and might explain, at least in part, the observed limitations in growth rate manifested as extended lag periods and lower proliferation rates. Indeed, ROS have been found to be highly efficient inhibitors of DNA and protein synthesis and can reduce the proliferation and hypertrophic growth of SCs and myoblasts [41,47,67,74]. ROS-induced insulin insensibility might be another reason for the observed decrease in growth [19,39,74].

L-Met restriction has been shown to decrease mitochondrial ROS production [40,41], whereas oversupplementation increases mitochondrial ROS generation [36]. With L-Met restriction, the concentrations of complexes I and IV of the respiratory chain decrease, and increased efficiency of complexes I and III (liver), as well as I (heart), has been found [41]. These investigations, however, have been performed after the isolation of mitochondria, a process that can have deleterious effects on its morphology and function [39].

In our present study, we investigated changes in the mitochondrial bioenergetics profile induced by supplementation with L-Met, DL-Met, or DL-HMTBA in intact C2C12 and QM7 cells, and therefore, mitochondria were kept in their physiological environment. In both cell types, oxidative phosphorylation (OXPHOS) was mainly (≈73%) used to generate the energy (ATP) needed, a finding also showing that most of the cells resembled activated, cycling myoblasts [73,75].

With the exception of a general reduction in PL after supplementation with methionine sources in C2C12 cells, bioenergetics parameters were not affected by L-Met or DL-Met supplementation. The PL, or “uncoupled respiration”, represented an outside–in H+ conductance (e.g., by mitochondrial uncoupling proteins; UCP 2 and 3) of the inner mitochondrial membrane that, thereby, regulated its membrane electrochemical gradient (proton motif force; Δψm) and, thus, ATP synthesis [76]. A decreased PL, as observed in C2C12 cells treated with any supplement, could thus reflect an adaptive response to stabilize Δψm. In accord, the Rmax, a measure of a cell’s ability to produce an electrochemical potential, was not affected by supplementation with any methionine source in C2C12 cells.

DL-HMTBA, however, affected several aspects of C2C12 and QM7 bioenergetics. In both cell types, load-dependent OCRbas was shown to be reduced at least in part due to the suppression of growth, resulting in lower consumption of ATP.

However, only in C2C12 cells was ATP-LR/%ATP-LR also decreased, and thus, a decrease in ATP synthase activity seemed to be the prominent response of C2C12 cells to oversupplementation with DL-HMTBA. The regulation of ATP-synthase activity is not completely understood, but its inhibition might result from (1) reduced activity or inhibition of respiratory chain enzymes [7,38], (2) changes in mTORC1 signaling [12,13], and (3) downregulation of its expression or functionality [47,77]. In various cells, a natural occurring regulator of mitochondrial ATP synthase, namely ATP synthase inhibitory factor 1 (IF 1), has been shown to exist, which specifically prevents the wasteful reverse mode of ATP-synthase (ATP hydrolysis) [78,79]. ATP-synthase suppression has been demonstrated to become especially important under conditions of cell stress [78,80], (1) to delay apoptosis, (2) to induce a phenotype that promotes proliferation, (3) to lower mitochondrial ROS (H_2_O_2_) production, and (4) to promote the switch from predominant mitochondrial ATP production to glycolysis [77,79,80,81]. In addition, lower OCRbas/ATP-LR values led to an increased ResC in DL-HMTBA-treated C2C12 cells by shifting the apparent respiratory state away from the Rmax, which has been shown to increase the resistance of muscle cells to mitochondrial dysfunction. Specifically, ROS-related insults can be tolerated for a longer period of time [82,83]. Such a mechanism helps to preserve a pool of healthy mitochondria, which is critical for cell survival and the maintenance of basal cell functions during acute and chronic stress. Likely, this is a reactive adaptive response to the higher OS in DL-HMTBA-treated compared with L-Met- and DL-Met-supplemented C2C12 cells.

In contrast to C2C12 cells, both the Rmax and ResC were reduced in QM7 cells supplemented with DL-HMTBA, pointing to reduced mitochondrial activity in response to OS. A reduced activity of the ROS-sensitive mitochondrial enzyme succinate dehydrogenase seemed to be involved, as we found a decrease in WST-1 reduction after treatment of QM7 cells with L-Met or DL-HMTBA [67]. Lower succinate production via the TCA cycle [84] could also contribute to this effect, which was masked during periods of higher metabolic activity, such as the active growth stage (around 72 h). However, different from L-Met-supplemented cells and in accordance with the higher stress level in DL-HMTBA-treated QM7 cells, nonmitochondrial respiration increased significantly in this group. By avoiding ROS production, nonmitochondrial aerobic glycolysis might be advantageous for fast-proliferating QM7 cells. Cells produce fewer ROS when they use glycolysis but more NADPH that must be re-oxidized with lactate dehydrogenase or via the trans-plasma membrane electron transport system (tPMET) [85]. Thus, the lower Rmax of DL-HMTBA-supplemented QM7 cells might reflect the increased amount of extramitochondrial respiration via tPMET.

Taking into account the higher proliferation and metabolic rate of quail QM7 cells, their ROS production should, theoretically, be higher than in mouse C2C12 cells. Interestingly, mitochondrial activity values as determined by WST-1 assay and extracellular H_2_O_2_, an indicator of OS level were both significantly lower in QM7 cells. In addition, only DL-HMTBA, which induced OS in both cell types, led to significant growth retardation in QM7 cells, whereas in C2C12 cells, L-Met and DL-HMTBA reduced growth to the same extent. Our data are in accordance with results from [57,86,87] showing that birds have a higher resistance to OS than mammals due to enhanced antioxidant defenses and a lower production of free oxygen radicals per unit of energy expended. Here, we showed that increased nonmitochondrial respiration (reduction in Rmax) and a reduction in overall mitochondrial activity (indicative of a higher proportion of aerobic glycolysis) contributed to the OS resistance of quail QM7 cells.

## 5. Conclusions

In conclusion, we found that the growth-promoting effect of the investigated methionine sources (L-Met, DL-Met, and DL-HMTBA) was concentration-dependent and resulted from positive effects on proliferation, cell viability and metabolic activity, and protein accumulation. As a first sign of oversupplementation, 1000 µM of L-Met (C2C12 cells) or DL-HMTBA (C2C12 and QM7 cells) retarded cell growth. However, only oversupplementation with DL-HMTBA induced OS, accompanied by changes in cellular bioenergetics, which differed between mouse C2C12 and quail QM7 cell lines.

As an adaptive response to prevent the reverse mode of ATP-synthase and, thus, ATP hydrolysis, ATP-synthase-linked respiration was decreased in C2C12 cells. In QM7 cells, the Rmax was reduced, but a strong increase in nonmitochondrial respiration was observed, indicating a switch to aerobic glycolysis. This could explain the lower increase in extracellular H_2_O_2_, a robust indicator of OS, in DL-HMTBA-supplemented QM7 compared with C2C12 cells. Therefore, we concluded that OS caused through high DL-HMTBA treatment could be better compensated for in quail cells, which is in accord with the literature showing higher OS resistance of bird cells [86,87].

The study had some limitations. For example, it was conducted in vitro and with mouse and avian cell lines only. Further studies might aim to verify the results in other species (e.g., humans or larger mammals), in primary muscle cells, or using ex vivo or in vivo approaches, which can consider biological variance between individuals as well. Due to low variation in experiments with cell lines, our sample size could be reduced to a minimum but should be increased when using primary cells or in vivo approaches.

Our results highlighted an important role of species differences in the ability to cope with methionine oversupplementation. Thus, before using amino acid supplementation in vitro or in vivo, the type and the concentration should be carefully selected and tested in order to benefit from desired positive effects and to avoid negative effects, e.g., due to oversupplementation.

## Data Availability

The data presented in this study are available on request from the corresponding author.
